# Emerging trends for urban freight transport–The potential for sustainable micromobility

**DOI:** 10.1371/journal.pone.0289915

**Published:** 2023-09-08

**Authors:** Jagienka Rześny-Cieplińska, Tomasz Tomaszewski, Maja Piecyk-Ouellet, Maja Kiba-Janiak

**Affiliations:** 1 Faculty of IT and Modern Technologies, WSB Merito University in Gdansk, Gdansk, Poland; 2 Faculty of Economics, University of Gdansk, Sopot, Poland; 3 Architecture and Cities, London, United Kingdom; 4 Department of Strategic Management and Logistics Wroclaw University of Economics and Business, Wroclaw, Poland; University of Hong Kong, HONG KONG

## Abstract

**Aim:**

Active transportation referring to non-motorized modes of transport is promoted and popularized both in practice and in the scientific literature, while their use for urban freight transport has been largely neglected. Thus the main scope of the paper is to indicate the development potential of micromobility use in urban freight transport and to check its influence on urban sustainability.

**Methods:**

The authors have hypothesized that active means of transport, with a focus on micromobility, have great development potential in freight transportation in cities. The implemented methods for analyzing the relationship between users’ characteristics, micromobility, and its impact on urban sustainable development, were logit and probit modelling. The authors’ system includes an analysis of factors connected with the topics of sustainability and micromobilty, that have met an essential scientific gap that this paper addresses.

Logistic (logit) regression is used mainly for binary, ordinal, and multi-level outcomes to find the probability of success (i.e. occurrence of some event). Probit regression, however, is primarily used in binary response models and assumes the normal distribution of data.

**Results:**

The main finding of the article has led the authors to the statement that active means of transport, including micromobility have great development potential in freight transportation in cities.

**Conclusions:**

Knowledge of the acceptance of micromobility solutions is essential for municipal authorities in shaping the development of urban transport systems. Thus proper strategies and actions need to be prioritized to leverage the sustainability-related co-benefits of active transport.

## 1. Introduction

The consequence of globalization and urbanization processes involves growing cities with a high accumulation and concentration of various economic activities. As a result, cities worldwide are facing challenges in developing sustainable, healthy, and efficient mobility. Contrary to people living in the country, city residents are much more exposed to numerous inconveniences like noise, harmful substances emissions, and congestion caused mainly by urban transport. Thus all urban transport stakeholders, significantly city inhabitants, could benefit from the practical solutions and infrastructure investments in providing new mobility models [1–4].

Research and reports on urban transport issues provide overviews of mobility solutions dedicated to passengers and freight that can improve sustainability and health outcomes [5–7] Especially sustainable mobility tends to be a key concept analyzed in the research literature [6–9]. Additionally, this is a practical problem because, recently, national policies have been focused intensively on sustainable development, environmental sustainability, and mobility in cities. Authorities at the local level realize that improving urban mobility should be crucial for the sustainable development of the cities. Thus numerous investments and incentives have been implemented to encourage city inhabitants to change their mobility habits and transportation choices [10–12]. While micromobility solutions classified as an active mode of transport used to be the subject of numerous research in the scientific literature, micromobility in urban freight transportation is rarely analyzed in the sources.

Accordingly, the article highlights the most important challenges related to the urban transport system with a focus on sustainable urban freight transport. The authors have analyzed how the micromobility solutions used for freight transport in cities contribute to achieving sustainability goals. The general aim of the paper was to indicate the possibilities and perspectives of sustainable urban freight transport development. To achieve this goal, the authors have used a survey to collect data from the respondents.The nextt stages were based on data analysis. The logit/probit method was used with the analysis which enables estimating connections between individual explanatory variables and determining their impact on the explained variable simultaneously.

The structure of the paper, was subordinated to the objectives. Firstly, researchers described the sustainability concept, next micromobility solutions were mentioned with their characteristics and main problems. In the following section, data, and methodology were presented with the subsequent research results and the conclusion containing research implications, limitations, and future research plans.

## 2. Literature review

### 2.1. Sustainability in cities

The concept of sustainability was initially mentioned during the 1980 World Conservation Strategy and was defined as a kind of development allowing the world’s ecosystems and biodiversity to stay sustainable. The 1987 Brundtland Report [13,14] (described sustainability more widely as “a kind of development that meets the needs of the present generations without compromising the ability of future generations to meet their own needs.” [15]. Then, during the UN Conference on Environment and Development in 1992 [15,16] in Rio, sustainability was finally defined as a multidimensional concept consisting of three pillars [17]:

· social equity;
· economic growth;
· environmental protection.

Considering sustainability development [18] as a three-dimensional notion, the question of whether these three factors are provided with equal support may be raised. International organizations and institutions indicate that the environmental issues [19] are prerequisite conditions for social justice and economic development [20–22].

Social sustainability [23,24] consists mainly of human health and resource security, aiming at preserving social capital by creating services that constitute the rules and framework of our functioning society. A sustainable business should be conducted following the ideas and support of employees and other stakeholders [25,26]. The approaches to providing this support may vary, but they should focus on treating employees fairly as significant community members, both locally and globally. Furthermore, this pillar of sustainability should focus on improving social quality by emphasizing honesty and the importance of relationships among people.

The main sustainable development principle addresses social and economic improvement that protects the environment and supports equality; therefore, the economy, society, and the ecological system are mutually dependent.

Economic sustainability [27,28] includes capital, effectiveness, and job creation, referring to the efficient use of assets to let the companies stay effective. The economic pillar of sustainability is where most businesses feel they are on firm ground to be sustainable; a business must be profitable. That said, profit cannot trump the other two pillars [28–32].

Environmental sustainability is defined as “a condition of balance, resilience and interconnectedness that allows human society to satisfy its needs while neither exceeding the capacity of its supporting ecosystems to continue to regenerate the services necessary to meet those needs nor by our actions diminishing bio-logical diversity” [26,33–37]. Providing clean water, clean air, or productive and clean land should be the basis of a responsible socioeconomic system. Furthermore, a sustainable production environment providing a raw material base is a prerequisite for building a sustainable society. The environmental area often gets the most attention. Organizations, institutions, and companies are focusing on reducing their carbon footprints, packaging waste, water usage, and their overall effect on the environment. Private entities have found that having a beneficial impact on the planet can also have a positive financial effect [38,39].

The three dimensions of sustainability [36,40–42] should be taken into consideration as equal because they complement to each other. For the company, being environmentally sustainable means being practical, e.g., using renewable materials.

Based on the original concept, the idea of sustainability in cities was developed [43–45]. According to the most current requirements, all mobility issues within the city areas [45–48] should be adapted to the sustainability rules [49–51]. The fact that numerous research has been conducted in regard to particular cities e.g. in China [52,53], Brazil [54] or based on worldwide experiences [47] is worth emphasizing.

### 2.2. Micromobility solutions

Micromobility is a relatively new solution but gradually (since 2020) starting to be analyzed in the scientific literature [55–59], with great potential for development. Micromobility allows city inhabitants to limit congestion and improve the quality of living in cities. This solution is commonly associated with the rapidly evolving range of light vehicles used for short-distance mobility within the urban area.

**Fig 1:**
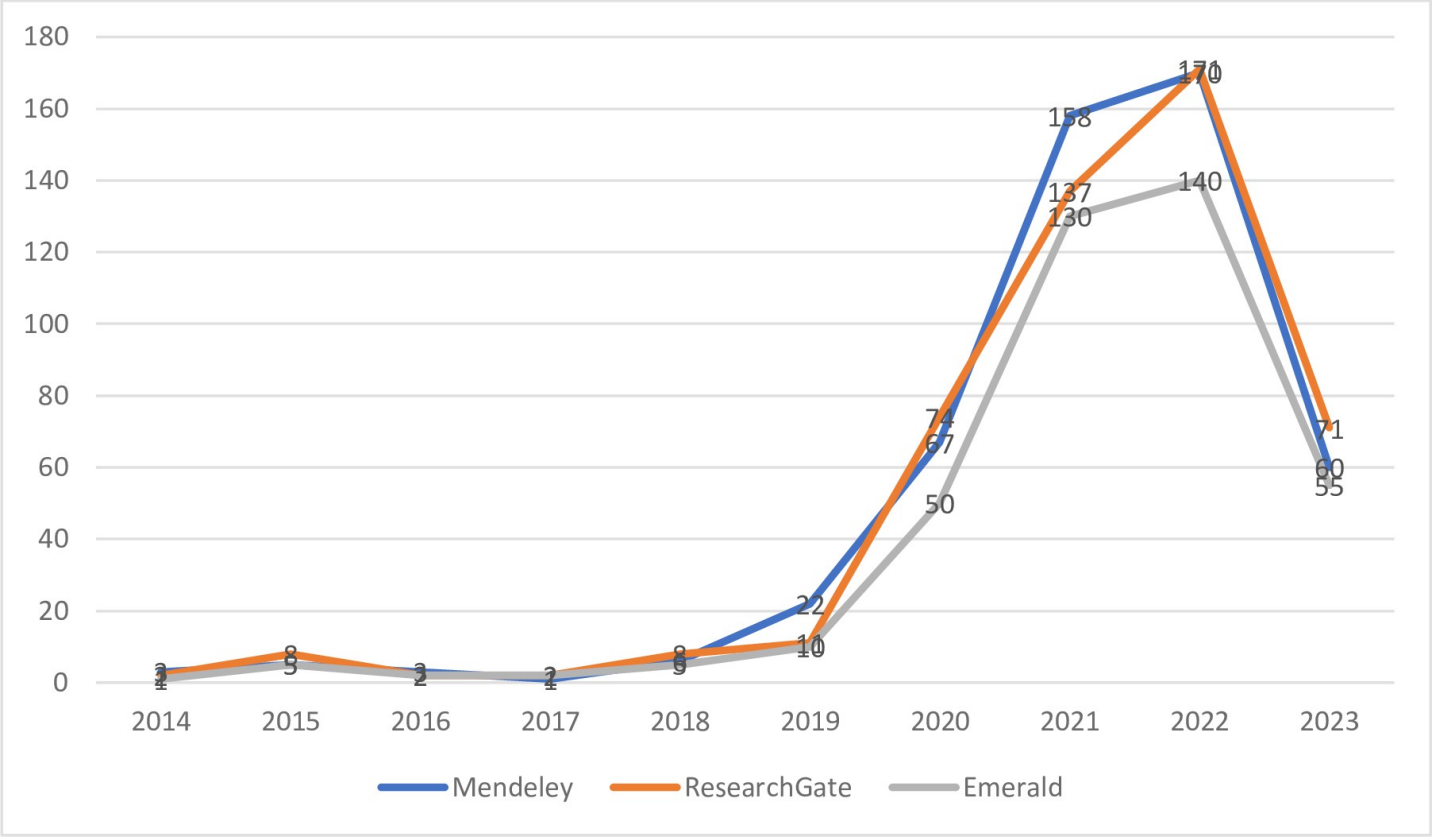
Micromobility in the literature review.

To conduct the literature review, the three databases were analyzed (Fig 1). According to the results, the interest in micromobility as a topic of papers was raised during the pandemic. This fact is a consequence of the behaviour of city authorities and city residents. The frequency of departures of public transport vehicles was reduced and owned and shared micro mobile vehicles ensured mobility around the city and social distancing.

As a term, micromobility was first used by H. Dediu, who described this way of transport as provided by shared vehicles weighing less than 500 kg. [60] This direction of analyzing micromobility has been reflected in numerous research papers. [55,56,61–63]. The International Transport Forum report called “Safe Micromobility” proposes classifying micromobility as “using micro vehicles weighing less than 350 kg with a speed of less than 45km/h.”

According to this definition, two types of vehicles represent micromobility–human-powered and electrically powered vehicles, e.g., to be more precise, IFT classifies micromobility vehicles into 4 groups:

- type A–unpowered or powered vehicles with speed up to 25 km/h and weight of 35 kg (bicycles, e-bikes);
- type B–vehicles with speed up to 25 km/h and weight of 35–350 kg;
- type C–powered vehicles with speeds up to 25–45 km/h and a weight of 35 kg;
- type D–vehicles with speeds up to 25–45 km/h and weight of 35–350 kg.

Regulatory frameworks related to micromobility vary depending on the country of origin. European Union regulations related to micromobility were established in law N°168/2013 [64]. L-category vehicles were specified and described as powered 2,3 and 4 wheels vehicles, using the criteria as power, speed, length, and weight. In two categories within this regulation, two examples of micromobility vehicles were included: electric bicycles (with speeds of up to 25 km/h and power between 250-100-W) and two-wheel cars (with speed of 25-45km/h and power to 4000W).

The efforts to classify micromobility were made in the US by SAE International [64]–a standard-developing organization. According to their assumptions, micromobility has been defined within the following criteria:

- top speed of 48km/h;
- weight of the vehicle up to 227 kg;
- width of the vehicle up to 1,5m;
- power sources: electric motor or combustion engine.

The New Urban Mobility Alliance NUMO [65] presents an exciting approach to micromobility issues. Besides typical criteria such as weight, power source, and top speed, emissions, spatial footprint, and health footprint are included. NUMO’s participants are cities, companies, and NGOs. Thus its recommendations are intended to help policymakers to plan urban transport strategies.

Considering various systematizations of micromobility vehicles (Table 1), the main criteria were taken into consideration including power sources (unpowered, human-powered, and electrically powered), shared or private (in the conducted research. On the basis of the literature review and previous research conducted by the authors, a list of sustainability criteria related to micromobility in cities was identified (Table 2).

**Table 1:** Types of micromobility vehicles.

Power source	unpowered/powered		powered	
Micromobility vehicles	TYPE A	TYPE B	TYPE C	TYPE D
Top speed	>25km/h		24-45km/h	
Weight	>35 kg	35-350kg	>35kg	35-350kg

**Table 2:** Sustainability criteria related to micromobility.

	Sustainability criteria	
Environmental	Social	Economic
1. Reduction emission of harmful substance 2. Reduction of noise 3, Less biodiversity waste 4. Using ecological resources 5. Reducing congestion	6. Safety on the road 7. Health benefits 8. Reduction of road accident 9. Better quality of life	10. Using resources effectively 11. Costs saving 12. Flexibility and accessibility 13. Time-saving

Thus the micromobility solutions were analyzed from the perspective of their sustainable character in environmental, social, and economic terms. Next, the particular criteria based on the literature sources were described in Table 3.

**Table 3:** Environmental, social and economic criteria related to micromobility–literature review results.

**Environmental criteria**	**Description**	**Sources**	
Reduction emission of harmful substance Reduction of noise Less biodiversity waste Using ecological resources Reducing congestion	Using low- or zero-emissions means of transport for urban mobility. Lower emissions of carbon dioxide (CO2) lead to a lower risk of climate change, and lower emissions of carbon monoxide (CO), SO2 sulphur dioxide, and nitrogen oxides (NO) lead to better air quality. Choosing quiet means of transport e.g. active means of transport such as bikes, electric vehicles, and public transport, recording and controlling noise, and keeping users informed about its level. Fewer environmental losses are caused by decreasing the use of individual modes of transport, especially a car. Using energy that produces no greenhouse gas emissions from fossil fuels and reduces some types of air pollution. Traffic reduction through promoting public transport or active means of transport for urban mobility.	[43,66] [67,68] [69] [70,71] [72,73]	
**Social criteria**		
Safety on the road Health benefits Reduction of road accidents Better quality of life	Ensuring the security of goods to be delivered. Choosing a mode of transport that can improve air quality and lead to better health outcomes. Fewer road traffic accidents by making traffic participants aware of driving customs to avoid accidents. Reduction of external costs related to transport.	[74] [75] [76,77] [78]
**Economic criteria**		
Using resources effectively Costs saving Flexibility and accessibility Time-saving	Effective use of transport means, infrastructure, and active transport means. Activities aimed at reducing the use of non-renewable resources and restoring renewable resources A range of possibilities is achieved by transporting goods every time and every place, to every route needed The most important factor	[79–81] [80,81] [58,62] [82,83]

## 3. Materials and methods

### 3.1. Research framework

In the study, three potential subjects have been addressed. First, regarding the sustainability criteria mentioned above for micromobility, the authors have decided to check whether the micromobility solutions fulfill the requirements of sustainable development. The relationship between sustainability and micromobility solutions has been emerging in the literature on the subject. However, it is often addressed on a theoretical level in general [84] or with scarce evidence regarding the COVID-19 impact [85].

The research process was designed in three layers: descriptive, methodological, and analytic. Firstly an initial literature review was conducted in order to identify the research gap. Secondly, the research scope and research questions were formulated, and the methodological approach was chosen on their basis,. During this phase, possible interrelationships between agreement with sustainability criteria (perception of passengers) and usage of micromobility transport facilities have been examined. Next, typical characteristics of passengers (e.g. gender, age, education, employment, and relationship status) and their perception of the usage of micromobility solutions have been taken into account, as in studies in different countries (especially–developed economies), e.g., Awad-Núñez et al. [86] Following this, as the data on different users of micromobility means of transportation were collected, the authors decided to prepare an analysis for urban freight transport and address the lack of scientific empirical papers in this area, taking into consideration factors such as individual characteristics of passengers, their perception of micromobility solutions, and usage of means of transportation.

On the basis of these steps, the research questions (RQ) were formulated:

RQ1: How do the micromobility solutions meet the criteria of sustainable development?

RQ2: Which individual characteristics of significantly different passengers determine the usage of micromobility means of transportation?

RQ3: How do the characteristics of passengers, usage of modes of transportation, and perception of micromobility solutions determine the use of micromobility means of transportation in urban freight transport?

Following this, the interview questionnaire was prepared and conducted.

**Fig 2:**
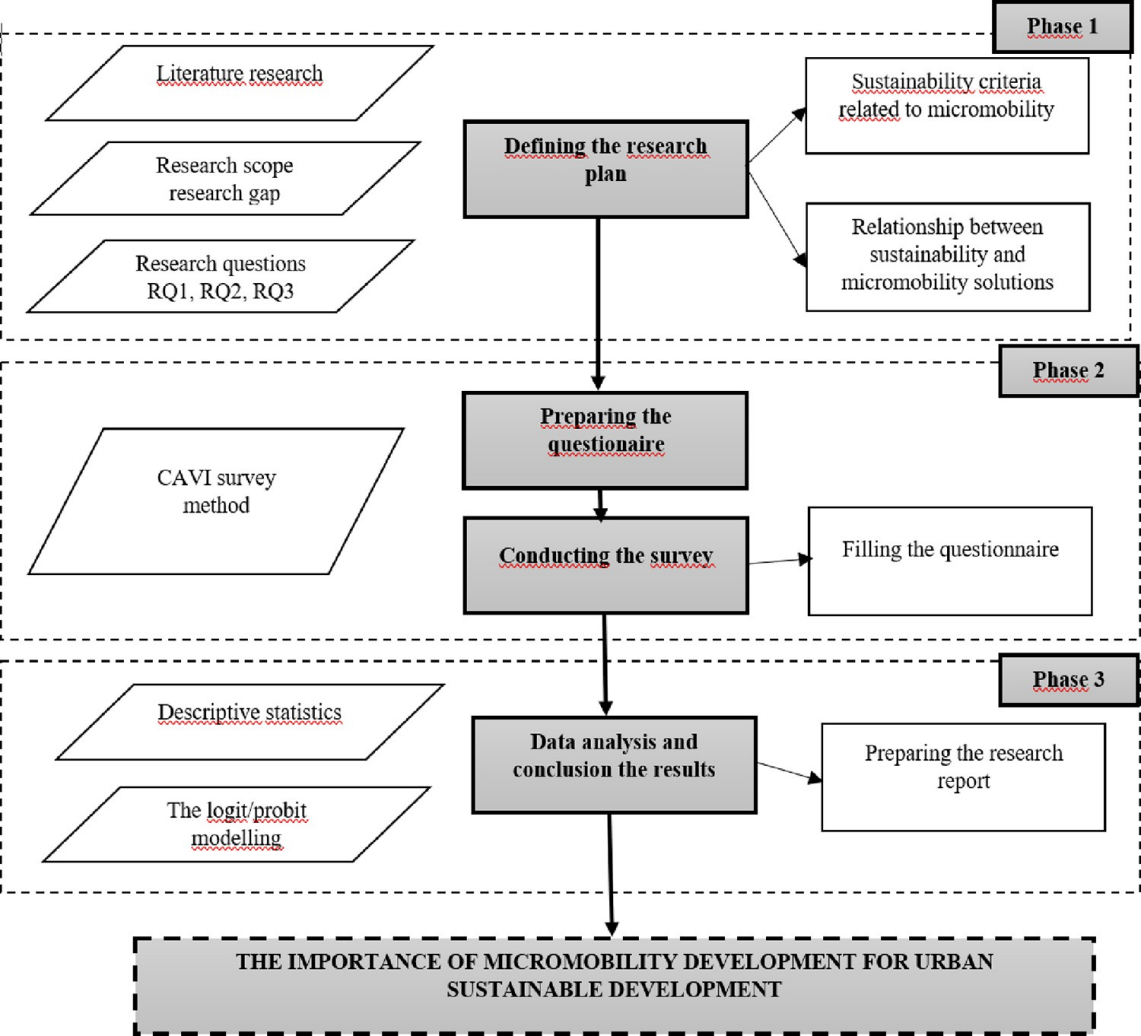
Research framework.

The next stages were based on data analysis. The logit/probit method was used with the analysis which enabled estimating connections between individual explanatory variables and determining their impact on the explained variable simultaneously (Fig 2).

### 3.2. Data collection

The authors collected data in the last months of 2021 based on the CAWI survey data with a random sample of over 500 Polish residents. For the research, the data collected by preparing a questionnaire were used. As a result, the authors obtained accurate information on 551 users of different means of transportation. The sample is representative as it considers the distribution of age in population, gender, and place of residence of potential passengers. One of the main features of such a database is unique information about micromobility features, usage of specific micromobility solutions by passengers, and perception of users of micromobility solutions on meeting sustainable development criteria, which are divided into categories.

As the data collected are based on a questionnaire, they are on different scales, such as continuous (e.g., *age*), binary (e.g., *work* status), ordinal (e.g., *income*) variables, and categorized in the Likert scale (e.g., sustainable development criteria). Descriptive statistics for all variables used in the study are presented in Appendix A and in basic form in Table 4.

**Table 4:** Descriptive statistics for the research sample.

Characteristics	Descriptive Statistics
Gender	Female 51,19% Male 48,27% Other 0,54%
Age	16–20 years old 10,88% 21–39 years old 50,83% 40–55 years old 38,29%
Place of residence	City over 100M of residents 30,30% 50-100M of residents 10,525% Below 50M of residents 19,41% Village with public transport access 19,60% Village w/o public transport access 20,14%

### 3.3. Data analysis

Since it may be sensitive to many different factors, the method of analysis should be selected in a careful manner. In literature, one can find numerous other ways to assess the relationships between micromobility and public transportation, such as the usage of multinomial logit models [87], negative binomial modelling [88], binomial logit modelling [89], binary logit modelling [90,91], Poisson regression [92], and analysis of descriptive statistics [93]. As the authors observe, many of those studies rely on logit modelling as a quite robust econometric approach; thus, regarding formulated hypotheses that are mainly discrete, the authors decided to prepare an analysis using logit/probit modelling. However, the authors’ system includes an analysis of factors connected with the topics of sustainability and micromobilty, and there is an essential scientific gap that this paper addresses.

Logistic (logit) regression is used mainly for binary, ordinal and multi-level outcomes to find the probability of success (i.e. the occurrence of some event). Probit regression, however, is primarily used in binary response models and assumes the normal distribution of data.

For example, in terms of logit regression, the dependent variable can be defined as:

$$•\;logit(p_{i})=\beta_{0}+\beta_{1}X_{1i}+\beta_{2}X_{2i}+\ldots .+\beta_{k}X_{ki}+\xi_{i},\;where\;X_{ni}’s\;refer\;to\;specific\;regressors,\;and$$ (1)

$$•\;\mathrm{logit}(p_{i})=\ln\frac{p_{i}}{1-p_{i}},\;\mathrm{where}\;p_{i}-\mathrm{probability}\;\mathrm{of}\;\mathrm{success}\;(1-p_{i}-\mathrm{probability}\;\mathrm{of}\;loss).$$ (2)

Following the described approach and formulated hypotheses, the authors calculated models accordingly. Firstly, regarding micromobility solutions and sustainable development, the authors checked the distribution of answers given by passengers on their agreement with the questions referring to the consistency of micromobility and sustainable development criteria.

Secondly, logit regression was utilised in terms of usage of micromobility facilities in general. The primary dependent variable is *use_micro_any* (discrete, binary) which refers to the fact that a passenger used any micromobility means of transportation (1 –yes, 0 –no). The authors applied logit regression to obtain the probability of using any micromobility means of transport by a specific person. Regarding control variables, the individual characteristics of passengers (*gender/sex*, *age* [in logarithmic scale], *resid* [place of residence–village, small town, large town etc.], *inc_pc* [income per capita], *edu* [educational status], *rel* [relationship status]), and working status), and passengers’ perception on using micromobility (e.g. existence of *gender gap* and types of ownership/sharing micromobility) have been taken into account.

Thirdly, when focusing on urban freight transport, the authors have included factors determining the usage of micromobility from the second step with the addition of currently used modes of transportation. The dependent variable is *use_aim_freight* with probit modelling as the normal data distribution is obtained. Thus, the probability of using micromobility in urban freight transport has been estimated.

## 4. Results

Table 3. depicts the distribution of responses regarding compliance with the requirements of sustainable development by micromobility solutions. The answers emphasizing the compliance of micromobility solutions with the criteria of sustainable development prevail in all categories of factors (economic, social, and environmental).

Therefore, taking into account the results presented in Table 5, one can conclude that the use of micromobility solutions by their users is significantly consistent with the perception of meeting the sustainability criteria by these measures. Thus, micromobility solutions meet the criteria of sustainable development.

**Table 5:** Sustainability criteria related to micromobility–distribution of results.

Sustainable development criteria	Engineless solutions	Electric engine solutions
1. Reducedemissions of harmful substances	• ‘no effect’– 14% • ‘small effect’– 9% • ‘moderate effect’– 15% • ‘substantial effect’– 12% • ‘crucial effect’– 50%	• ‘no effect’– 14% • ‘small effect’– 15% • ‘moderate effect’– 31% • ‘substantial effect’– 15% • ‘crucial effect’– 25%
2. Reduction of noise	• ‘no effect’– 12% • ‘small effect’– 9% • ‘moderate effect’– 19% • ‘substantial effect’– 16% • ‘crucial effect’– 44%	• ‘no effect’– 13% • ‘small effect’– 14% • ‘moderate effect’– 29% • ‘substantial effect’– 20% • ‘crucial effect’– 24%
3, Less biodiversity waste	• ‘no effect’– 15% • ‘small effect’– 13% • ‘moderate effect’– 25% • ‘substantial effect’– 15% • ‘crucial effect’– 32%	• ‘no effect’– 16% • ‘small effect’– 15% • ‘moderate effect’– 33% • ‘substantial effect’– 19% • ‘crucial effect’– 17%
4. Using ecological resources	• ‘no effect’– 14% • ‘small effect’– 11% • ‘moderate effect’– 23% • ‘substantial effect’– 18% • ‘crucial effect’– 34%	• ‘no effect’– 13% • ‘small effect’– 13% • ‘moderate effect’– 36% • ‘substantial effect’– 17% • ‘crucial effect’– 21%
5. Reducing congestion	• ‘no effect’– 12% • ‘small effect’– 10% • ‘moderate effect’– 26% • ‘substantial effect’– 20% • ‘crucial effect’– 33%	• ‘no effect’– 16% • ‘small effect’– 14% • ‘moderate effect’– 30% ‘substantial effect’– 18% ‘crucial effect’– 22%
6. Safety on road	• ‘no effect’– 16% • ‘small effect’– 16% • ‘moderate effect’– 32% • ‘substantial effect’– 19% • ‘crucial effect’– 17%	• ‘no effect’– 20% • ‘small effect’– 17% • ‘moderate effect’– 33% • ‘substantial effect’– 16% • ‘crucial effect’– 14%
7. Health benefits	• ‘no effect’– 7% • ‘small effect’– 10% • ‘moderate effect’– 22% • ‘substantial effect’– 21% • ‘crucial effect’– 40%	• ‘no effect’– 15% • ‘small effect’– 16% • ‘moderate effect’– 30% • ‘substantial effect’– 20% • ‘crucial effect’– 19%
8. Road accidents reduction	• ‘no effect’– 19% • ‘small effect’– 15% • ‘moderate effect’– 27% • ‘substantial effect’– 21% • ‘crucial effect’– 18%	• ‘no effect’– 21% • ‘small effect’– 16% • ‘moderate effect’– 32% • ‘substantial effect’– 17% • ‘crucial effect’– 14%
9. Better quality of life	• ‘no effect’– 15% • ‘small effect’– 13% • ‘moderate effect’– 27% • ‘substantial effect’– 22% • ‘crucial effect’– 23%	• ‘no effect’– 15% • ‘small effect’– 15% • ‘moderate effect’– 29% • ‘substantial effect’– 22% • ‘crucial effect’– 19%
10. Using resources effectively	• ‘no effect’– 13% • ‘small effect’– 14% • ‘moderate effect’– 28% • ‘substantial effect’– 25% • ‘crucial effect’– 20%	• ‘no effect’– 15% • ‘small effect’– 15% • ‘moderate effect’– 32% • ‘substantial effect’– 21% • ‘crucial effect’– 17%
11. Costs saving	• ‘no effect’– 9% • ‘small effect’– 12% • ‘moderate effect’– 27% • ‘substantial effect’– 23% • ‘crucial effect’– 29%	• ‘no effect’– 12% • ‘small effect’– 18% • ‘moderate effect’– 33% • ‘substantial effect’– 20% • ‘crucial effect’– 17%
12. Flexibility and accessibility	• ‘no effect’– 10% • ‘small effect’– 11% • ‘moderate effect’– 27% • ‘substantial effect’– 25% • ‘crucial effect’– 27%	• ‘no effect’– 10% • ‘small effect’– 10% • ‘moderate effect’– 31% • ‘substantial effect’– 24% • ‘crucial effect’– 25%
13. Time-saving	• ‘no effect’– 14% • ‘small effect’– 16% • ‘moderate effect’– 28% • ‘substantial effect’– 21% • ‘crucial effect’– 21%	• ‘no effect’– 13% • ‘small effect’– 12% • ‘moderate effect’– 27% • ‘substantial effect’– 21% • ‘crucial effect’– 27%

In the next step, the factors influencing the usage of micromobility, in general, were analyzed. As shown before, the authors considered the characteristics of the respondents (e.g. gender, age, education, level of income, number of people in the household, the form of employment) and variables related to the use and ownership of micromobility solutions. The results have been presented in Appendix B. The main determinants differentiating the results are gender and age (e.g. ceteris paribus, the older the people are, the less likely they are to use micromobility solutions). The results also differ for the income category, the number of people staying in the household, etc.

Finally, results for a specific type of transportation–urban freight transport were obtained. The results have been presented in Table 6.

**Table 6:** Probability of using micromobility means of transportation in urban freight transport–key determinants.

	(1)	(2)	(3)
Variables	General Model	Estimates—Women	Estimates—Men
mode_bus	-0.00523	-0.00835	-0.0146
	(0.00955)	(0.0217)	(0.0115)
mode_train	-0.0117	-0.0192	-0.0160
	(0.0139)	(0.0267)	(0.0153)
mode_car	-0.00504	-0.00512	-0.0194*
	(0.00923)	(0.0218)	(0.0110)
mode_mtrbike	0.00890	0.0628**	-0.0357**
	(0.0115)	(0.0310)	(0.0163)
mode_taxi	0.0121	0.0174	0.00859
	(0.0144)	(0.0256)	(0.0184)
mode_bike	0.00262	0.00259	-0.00851
	(0.0104)	(0.0254)	(0.0126)
mode_scooter	0.0846***	0.191**	0.0626*
	(0.0281)	(0.0808)	(0.0347)
mode_foot	0.00270	0.00789	-0.0175
	(0.00966)	(0.0213)	(0.0120)
2.gendersex	0.103		
	(0.149)		
3o.gendersex	-		
ln_age	0.625**	0.188	0.321
	(0.285)	(0.497)	(0.450)
2.resid	-0.572***	-0.827**	-0.382
	(0.213)	(0.342)	(0.356)
3.resid	-0.108	0.296	0.0346
	(0.208)	(0.356)	(0.318)
4.resid	-0.464*	0.0290	-0.562
	(0.239)	(0.369)	(0.398)
5.resid	-0.374*	0.0131	-0.891***
	(0.205)	(0.321)	(0.340)
2.housing_ppl	0.819**	1.229**	1.537***
	(0.336)	(0.571)	(0.525)
3.housing_ppl	1.001***	1.827***	1.038**
	(0.331)	(0.558)	(0.499)
4.housing_ppl	0.909**	1.778***	0.914
	(0.402)	(0.670)	(0.620)
housing_childu16	-0.273	-0.695**	-0.0564
	(0.180)	(0.330)	(0.294)
2.inc_pc	-0.382	-1.746***	0.000229
	(0.373)	(0.601)	(0.620)
3.inc_pc	-0.185	-0.420	0.0316
	(0.249)	(0.341)	(0.446)
4.inc_pc	-0.277	-0.929***	0.437
	(0.220)	(0.309)	(0.383)
5.inc_pc	-0.606***	-0.827**	-0.108
	(0.227)	(0.328)	(0.403)
6.inc_pc	-0.469**	-0.817**	-0.201
	(0.222)	(0.350)	(0.376)
7.inc_pc	-0.374	-0.321	-0.459
	(0.249)	(0.387)	(0.431)
8.inc_pc	-0.621*	-1.761***	0.243
	(0.345)	(0.595)	(0.520)
2.edu	-0.337	-0.123	-0.222
	(0.376)	(0.635)	(0.591)
3.edu	-0.730*	-0.240	-1.156
	(0.428)	(0.702)	(0.730)
4.edu	-0.458	-0.216	-0.476
	(0.375)	(0.682)	(0.571)
5.edu	-0.516	0.730	-1.519**
	(0.418)	(0.712)	(0.680)
6.edu	-0.244	0.299	-0.792
	(0.393)	(0.644)	(0.598)
7.edu	-0.341	-0.117	-0.104
	(0.379)	(0.638)	(0.581)
8.edu	-0.617	0.312	-0.939
	(0.482)	(0.738)	(0.773)
9o.edu	-		-
2.rel	0.623	1.819*	
	(0.646)	(0.964)	
3.rel	-0.524	-1.184*	-0.0623
	(0.398)	(0.651)	(0.644)
4.rel	-0.187	0.0512	-0.548
	(0.203)	(0.343)	(0.339)
5.rel	-0.0916	0.454	-0.0240
	(0.217)	(0.354)	(0.361)
work_prof	0.0120	-0.543	0.369
	(0.238)	(0.460)	(0.387)
work_notprof	0.644***	-0.128	1.722***
	(0.247)	(0.433)	(0.407)
work_prof_other	0.595**	0.270	1.185***
	(0.254)	(0.453)	(0.368)
work_selfempl	-0.191	0.155	-0.317
	(0.356)	(0.567)	(0.521)
work_studying	0.154	-0.419	0.174
	(0.269)	(0.530)	(0.447)
work_pens	0.318		0.437
	(0.689)		(0.812)
work_annuit	0.0149	-1.368*	0.576
	(0.389)	(0.813)	(0.621)
2.gender_gap	-0.118	-0.593	0.00570
	(0.265)	(0.483)	(0.363)
3.gender_gap	0.0690	-0.101	0.191
	(0.236)	(0.425)	(0.317)
4.gender_gap	-0.0853	-0.407	-0.614*
	(0.241)	(0.465)	(0.358)
5.gender_gap	-0.0362	-0.616	0.224
	(0.288)	(0.508)	(0.442)
2.micro_own_shared_both	0.0179	0.221	-0.280
	(0.194)	(0.336)	(0.304)
3.micro_own_shared_both	-0.251	0.106	-0.955***
	(0.160)	(0.233)	(0.303)
2.micro_use_shared_own_both	0.0748	0.442	-0.199
	(0.191)	(0.290)	(0.300)
3.micro_use_shared_own_both	-0.0539	0.447	-0.687**
	(0.201)	(0.298)	(0.347)
micro_use_alone	0.0301	0.0744	-0.0698
	(0.156)	(0.250)	(0.260)
micro_use_shared_ppl	0.101	0.0353	0.231
	(0.152)	(0.258)	(0.243)
micro_use_kids	0.0925	0.106	0.113
	(0.183)	(0.286)	(0.322)
micro_use_anm	0.0188	-0.980**	0.504
	(0.272)	(0.445)	(0.408)
2.use_sugg_micro	0.218	0.178	0.148
	(0.174)	(0.273)	(0.286)
3.use_sugg_micro	-0.281	-0.659**	0.280
	(0.172)	(0.279)	(0.260)
*Constant*	-2.598*	-1.810	-0.467
	(1.376)	(2.640)	(1.959)
Observations	547	282	263
Wald chi2	104.31	104.68	84.47
Prob > chi2	0.0001	0.0001	0.0065
Pseudo R-squared	0.1687	0.3166	0.2784

Robust standard errors.

*** p<0.01

** p<0.05

* p<0.1.

For the general model, the critical differentiating factors include age (ceteris paribus, the older the person is, the more willingly s/he uses micromobility to transport goods), the number of people in the household (the more people residue in the house, the more likely they use micromobility—results also hold for both men and women), the level of income (ceteris paribus, the higher the level of earnings, the statistically lower the probability of using micromobility—results also relevant to women) and the type of employment (ceteris paribus, people employed on contracts are more likely to use micromobility to transport goods). Notably, the factors related to the perception of micromobility are statistically insignificant here.

## 5. Discussion and limitations

The paper consists of a few subjects, and first of all investigates the impact of micromobility solutions on sustainability level, and subsequently, the relationships between the passengers’ characteristics and the propensity to use micro mobile vehicles for freight transport.

Our study supports the position, that micromobility solutions meet the criteria of sustainable development and this is the first study to compare the micromobility used for UFT’s impact on the sustainable development of cities. This is somewhat surprising given the fact that in recent years the papers from scientific journals have focused on the micromobility potential quite intensively. However, the mainstream scientific literature agrees as to the role of these new modes (classified as micro mobile vehicles) in sustainable development [94–96], analyzing the subject in the general sense [94] or in relation to passengers’ mobility [57,63].

Existing research is used to build a three-pillar approach, that is then implemented for evaluating the extent to which micromobility contributes to the sustainability of urban transport systems. Nevertheless, the environmental performance of micromobility solutions is quite frequently the subject of the analyses, where the authors calculate the impact of micromobility on the emissions of harmful substances [97], congestion [98], or generation of other external costs [99]. As regards the relationship between the probability of using micromobility modes of transportation (in general and in relation to urban freight transport) and the individual characteristics of passengers, our research results have confirmed the approaches met in the literature sources [100–103].

Although the authors, while preparing and conducting the research, made an effort to ensure that the results obtained were as plausible and credible as possible, they have been aware of certain limitations related to the study. The COVID-19 pandemic significantly reduced travel in general and disrupted travel patterns across cities all over the world, affecting urban mobility which was confirmed in reports [94] and scientific research [85,104,105]. After an initial dip, micromobility services, including the shared ones, have gained popularity as ensuring social distancing. Previous research has compared data before and after the pandemic, providing relevant conclusions that could help authorities plan future policies and improve the infrastructure needed to promote micromobility services. A possible limitation of our study comes from, the fact that our research was conducted, and the findings have been based on the survey of micromobility users in Polish cities during the second phase of the COVID-19 pandemic. Overall, the COVID-19 pandemic acted as a catalyst for the adoption and transformation of micromobility. It highlighted the importance of flexible, sustainable, and individualized transportation options, leading to changes in infrastructure, usage patterns, and regulations to accommodate the evolving needs of communities. Numerous changes in micromobility have been observed: increased popularity, expansion of bile lanes, adoption of shared mobility services, integration with public transportation and regulatory changes related to the micromobility solutions. Thus, the authors’ intention was to present how the behaviours of the users have changed, in long-time perspective.

Secondly, the results for one country have been presented, and collecting and analyzing data from different countries would give a broader picture of the use of micromobility for passenger and freight transport. And finally, the respondents were asked if the micromobility met the particular sustainability criteria. In future work, more factors should be taken into consideration including e.g. no battery recycling issues, legal restrictions on movements, and accidents with other road users, to name a few.

## 6. Conclusion and future research

The use of micromobility solutions as an additional mode in urban transport systems begins to be a component of transport behaviors. Since micromobility used in passenger transport is undoubtedly expected to grow in the future, our study has examined this mode’s share in UFT. Therefore the analysis consisted of a few subjects—investigating whether micromobility met Poland’s sustainable development criteria and examining the relationship between the probability of using micromobility modes of transportation (in general and urban freight transport) and the individual characteristics of passengers. While the impact of the use of micro mobile vehicles on sustainable development has been confirmed and accepted due to the consistency of the results obtained from the analysis of passenger responses, the relationship between the characteristics of the respondents and their propensity to use modern modes is a more complex problem. Results for using micromobility in urban freight transport, confirming the leading role of age, income, and housing conditions as the main differentiating factors in the analysis, were estimated. Yet, one should underline the scarce evidence on the impact of the perception on sharing micromobility on the usage of this type of transportation facility.

The current analysis points out the directions for further research:

1. The study should be deepened by conducting repetitive cross-section models in the future to prepare for sensitivity analysis.
2. This kind of research would enable one to show much more differentiated results regarding the probability of using micromobility solutions and meeting sustainability criteria for micromobility.
3. Comparing micromobility transportation modes (and assessing the likelihood of their use) in different countries would be an asset.

Secondly, however, the obtained cross-section data have not allowed us to conduct a more detailed analysis regarding key determinants of urban freight modes of transportation because of the relatively low number of observations for specific micromobility solutions. Therefore, there should be an effort made to collect more targeted data on urban freight modes of transportation using micromobility to have the analysis significantly enriched.

As addressed above by the authors, there is plenty of room for changes in the perception of micromobility in Poland, indicating the directions and areas of future research, so it is hoped that this study will continue the scientific discussion about micromobility development and its share in urban mobility, both in passenger and freight transportation Passengers have been increasingly likely to use new ways of transportation—those related to micromobility vehicles in general, as well as micromobility for urban freight transport. What is crucial is that, it is followed by their increasing awareness of meeting sustainable development goals through these modes of transportation. Of course, people differ a lot, and these differences significantly determine their use of micromobility. However, one important conclusion is viable, namely, there is vast, initially exposed the potential for the usage of sustainable means of transportation that would lead to a significant shift toward micromobility solutions.

## Supporting information

S1 Table Descriptive statistics for variables used in the study–access on Open Science Framework. (PDF)

S2 Table Estimation of probability of using micromobility means of transportation–key determinants—access on Open Science Framewo. (PDF)

S3 Table Questionnaire results. (XLS)
