# Supplementary material for: Emerging trends for urban freight transport–The potential for sustainable micromobility
Source: PLoS One. 2023 Sep 8;18(9):e0289915. doi: 10.1371/journal.pone.0289915 (PMC10490950; doi:10.1371/journal.pone.0289915)
Supplement: S1 Table — (PDF) [file pone.0289915.s001.pdf]

Appendix A

Descriptive statistics for variables used in the study

Variable	Obs	Mean	Std. Dev.	Min	Max
gendersex	551	1.493648	0.5111978	1	3
resid	551	3.112523	1.520182	1	5
mode_bus	551	17.25045	26.07191	0	100
mode_train	551	2.882033	8.750203	0	100
mode_car	551	50.6225	35.93736	0	100
mode_mtrbike	551	1.205082	6.841827	0	100
mode_taxi	551	2.061706	6.745221	0	80
mode_bike	551	6.257713	12.17594	0	100
mode_scooter	551	0.6206897	2.572485	0	30
mode_foot	551	18.11252	20.51133	0	100
use_aim_shopping	551	0.2286751	0.4203612	0	1
micro_own_shared_both	551	2.179673	0.8833946	1	3
micro_use_shared_own_both	551	2.14882	0.7529297	1	3
micro_use_alone	551	0.5372051	0.4990669	0	1
micro_use_shared_ppl	551	0.5027223	0.5004469	0	1
micro_use_kids	551	0.2032668	0.4027951	0	1
micro_use_anm	551	0.0653358	0.2473419	0	1
use_sugg_micro	551	2.176044	0.820115	1	3
micro_sustdevreq_engineless_emco2	551	3.744102	1.496006	1	5
micro_sustdevreq_engineless_noise	551	3.696915	1.413175	1	5
micro_sustdevreq_engineless_biodiv	551	3.362976	1.426633	1	5
micro_sustdevreq_engineless_resuse	551	3.459165	1.413462	1	5
micro_sustdevreq_engineless_congestion	551	3.524501	1.343956	1	5
micro_sustdevreq_engineless_safety	551	3.039927	1.298336	1	5
micro_sustdevreq_engineless_health	551	3.758621	1.274146	1	5
micro_sustdevreq_engineless_redtraffcoll	551	3.054446	1.355371	1	5
micro_sustdevreq_engineless_livstand	551	3.257713	1.341237	1	5
micro_sustdevreq_engineless_useinfra	551	3.255898	1.274162	1	5
micro_sustdevreq_engineless_costredusers	551	3.491833	1.277578	1	5
micro_sustdevreq_engineless_availability	551	3.484574	1.266074	1	5
micro_sustdevreq_engineless_savetime	551	3.186933	1.327846	1	5
micro_sustdevreq_engine_emco2	551	3.239564	1.345955	1	5
micro_sustdevreq_engine_noise	551	3.30127	1.31425	1	5
micro_sustdevreq_engine_biodiv	551	3.049002	1.287476	1	5
micro_sustdevreq_engine_resuse	551	3.210526	1.274633	1	5
micro_sustdevreq_engine_congestion	551	3.172414	1.344768	1	5
micro_sustdevreq_engine_safety	551	2.885662	1.298808	1	5
micro_sustdevreq_engine_health	551	3.103448	1.310866	1	5
micro_sustdevreq_engine_redtraffcoll	551	2.849365	1.313218	1	5
micro_sustdevreq_engine_livstand	551	3.130672	1.309123	1	5
micro_sustdevreq_engine_useinfra	551	3.098004	1.282546	1	5
micro_sustdevreq_engine_costredusers	551	3.136116	1.231991	1	5
micro_sustdevreq_engine_availability	551	3.446461	1.24036	1	5
micro_sustdevreq_engine_savetime	551	3.362976	1.353383	1	5
gender_gap	551	3.127042	1.125819	1	5
work_notprof	551	0.1742287	0.3796507	0	1
work_prof	551	0.5880218	0.4926384	0	1
work_prof_other	551	0.0871143	0.2822588	0	1
work_selfempl	551	0.0671506	0.2505101	0	1
work_pens	551	0.0090744	0.0949127	0	1
work_annuit	551	0.030853	0.1730764	0	1
work_studying	551	0.1415608	0.3489159	0	1

housing	551	4.800363	1.263502	1	7
rel	551	3.689655	1.607896	1	5
housing_ppl	551	2.745917	0.6747772	1	4
housing_childu16	551	0.5480944	0.4981338	0	1
inc_pc	551	4.437387	2.106954	1	8
edu	551	4.715064	2.053939	1	9
use_micro_any	551	0.6116152	0.4878257	0	1
age	551	35.19056	10.99082	16	55

Source: Own elaboration.
