# Supplementary material for: Emerging trends for urban freight transport–The potential for sustainable micromobility
Source: PLoS One. 2023 Sep 8;18(9):e0289915. doi: 10.1371/journal.pone.0289915 (PMC10490950; doi:10.1371/journal.pone.0289915)
Supplement: S2 Table — (PDF) [file pone.0289915.s002.pdf]

Appendix B

Estimation of probability of using micromobility means of transportation – key determinants

**Logistic regression**			**Number of obs.**	**549**
			Wald chi2(50)	116.64
			Prob > chi2	0.0000
Log likelihood = -282.11229			Pseudo R2	0.2315
use_micro_any	Coef.	Std. Err.	z	P>z
gendersex				
2	0.502**	0.237	2.12	0.034
3	0.204	1.273	0.16	0.873
ln_age	-2.829	0.477	-5.93	0.000
resid				
2	0.654*	0.353	1.85	0.064
3	0.146	0.335	0.44	0.663
4	0.800*	0.409	1.95	0.051
5	0.852***	0.320	2.66	0.008
housing_ppl				
2	0.854*	0.485	1.76	0.078
3	1.230**	0.481	2.55	0.011
4	2.165***	0.672	3.22	0.001
inc_pc				
2	0.182	0.766	0.24	0.812
3	0.713*	0.421	1.69	0.091
4	0.039	0.376	0.10	0.917
5	0.253	0.376	0.67	0.501
6	0.141	0.345	0.41	0.682
7	0.525	0.395	1.33	0.184
8	-0.243	0.584	-0.42	0.677
edu				
2	1.641**	0.645	2.54	0.011
3	1.157*	0.662	1.75	0.081
4	1.036*	0.627	1.65	0.099
5	1.527**	0.701	2.18	0.029
6	0.606	0.643	0.94	0.346
7	1.587**	0.628	2.52	0.012
8	1.405*	0.774	1.82	0.069
rel				
2	1.522*	0.798	1.91	0.057

3	-0.207	0.577	-0.36	0.720
4	-0.081	0.320	-0.25	0.800
5	0.077	0.313	0.25	0.806
work_prof	0.770*	0.397	1.94	0.053
work_not_prof	0.689	0.439	1.57	0.116
work_prof_other	1.299***	0.460	2.82	0.005
work_selfempl	0.303	0.536	0.57	0.571
work_studying	-0.043	0.437	-0.10	0.920
work_pens	1.706	1.319	1.29	0.196
work_annuit	-0.730	0.768	-0.95	0.342
gender_gap				
2	1.083**	0.438	2.47	0.013
3	0.221	0.381	0.58	0.561
4	1.128***	0.403	2.80	0.005
5	0.224	0.470	0.48	0.633
micro_own_shared_both				
2	-0.562*	0.333	-1.69	0.092
3	-1.39***	0.299	-4.66	0.000
micro_use _shared_own_both				
2	-0.471	0.312	-1.51	0.131
3	0.302	0.337	0.90	0.371
micro_use_alone	-0.306	0.257	-1.19	0.235
micro_use_shared_ppl	-0.157	0.249	-0.63	0.527
micro_use_kids	-0.348	0.267	-1.30	0.192
micro_use_anm	0.023	0.452	0.05	0.958
use_sugg_micro				
2	0.248	0.295	0.84	0.401
3	-0.292	0.266	-1.10	0.271
_cons	7.203	1.768	4.07	0.000
